# Mothers’ perceptions and decision-making regarding tandem breastfeeding in Türkiye: a child health-focused qualitative study

**DOI:** 10.1186/s12884-026-09764-0

**Published:** 2026-08-03

**Authors:** Meltem Aslan, Cemile Say, Hilal Ezgi Navruz, Şimal Niğiz, Zeliha Özge Kuroğlu

**Affiliations:** 1 Department of Midwifery, Faculty of Health Sciences, Istanbul Esenyurt University, Istanbul, Türkiye; 2 Department of Nursing, Faculty of Health Sciences, Istanbul Esenyurt University, Istanbul, Türkiye

**Keywords:** Tandem breastfeeding, Qualitative research, Breastfeeding, Motherhood, Child health

## Abstract

**Background:**

Tandem breastfeeding, defined as breastfeeding two non-twin children during the same period, remains an underexplored practice in maternal and child health. Although it may be perceived as a way of maintaining breastfeeding continuity and emotional closeness with the older child, mothers may also encounter physical, emotional, practical, social and professional challenges. This study aimed to explore mothers’ perceptions of tandem breastfeeding in Türkiye and to examine how these perceptions are shaped by emotional, socio-cultural, practical and healthcare-related factors.

**Methods:**

A qualitative study design based on reflexive thematic analysis was used. Semi-structured, in-depth interviews were conducted with 21 mothers who had at least one child aged 0–2 years and were familiar with the concept of tandem breastfeeding. Mothers were recruited using purposive and snowball sampling. Current or previous direct tandem breastfeeding experience was not systematically recorded as a mother characteristic. Interviews were audio-recorded, transcribed verbatim and analysed inductively using MAXQDA 24. The study was reported in accordance with the COREQ checklist.

**Results:**

Four main themes were identified: the perceived meaning of tandem breastfeeding, the perceived burden of tandem breastfeeding, social perceptions, and limited professional support. Mothers associated tandem breastfeeding with maintaining the mother–child bond, preventing abrupt weaning of the older child, and supporting a sense of fairness between siblings. They also perceived it as potentially involving physical fatigue, sleep deprivation, emotional ambivalence, increased care demands, and uncertainty regarding practical feasibility. Social norms, family attitudes, misconceptions, and limited access to evidence-based professional guidance influenced mothers’ decision-making. Because direct tandem breastfeeding experience was not systematically recorded, the findings represent a combination of perceptions, beliefs, observed examples, anticipated concerns, and decision-making considerations rather than the confirmed lived experiences of a clearly defined group of tandem breastfeeding mothers.

**Conclusions:**

Tandem breastfeeding was perceived as a complex maternal and child health practice shaped by emotional, practical, socio-cultural, and healthcare-related factors. The findings provide insight into how mothers understand and evaluate tandem breastfeeding and how they approach decisions about whether to initiate or continue the practice. However, because mothers with confirmed direct tandem breastfeeding experience could not be distinguished from those expressing perceptions, expectations, or observed experiences, the findings should not be interpreted as evidence of the lived experience, feasibility, safety, or clinical outcomes of tandem breastfeeding. Further research involving clearly identified mothers with direct tandem breastfeeding experience is needed.

## Introduction

Breast milk is the ideal food for infants, as it is clean and safe and contains antibodies that help protect against childhood illnesses. The World Health Organisation (WHO) recommends that infants be exclusively breastfed for the first 6 months to achieve optimal growth, development and health, and that breastfeeding be continued alongside nutrient-rich complementary foods until the age of 2 or beyond [1, 2].

Breastfeeding two non-twin siblings simultaneously is known as tandem breastfeeding [3]. The American Academy of Family Physicians states that breastfeeding during a subsequent pregnancy is not unusual and that, when the pregnancy is normal and the mother is healthy, continuing breastfeeding is a personal decision. The AAFP also notes that breastfeeding beyond infancy may continue as long as mutually desired [4]. Mothers may become pregnant again whilst breastfeeding, and in such cases, they may feel uncertain about whether to continue breastfeeding. When the new baby is born, this uncertainty may become more complex, particularly because tandem breastfeeding mothers may experience a stronger perception of breast milk insufficiency. Erdoğan and Turan reported that mothers who continued breastfeeding during pregnancy had a more intense perception of insufficient milk than mothers breastfeeding only one child; however, their study did not specifically evaluate weaning decisions or distinguish whether the older child was fed through direct breastfeeding, expressed breast milk, or both [5]. Furthermore, mothers who practise tandem breastfeeding are often subject to criticism from those around them. Nevertheless, it is known that there are mothers who choose tandem breastfeeding [6].

Informing and encouraging mothers to continue breastfeeding for up to two years is among the responsibilities of nurses and midwives. However, the lack of guidance materials on tandem breastfeeding and the resulting information gap lead to inadequate counselling for mothers [7].

Despite increasing interest in breastfeeding continuity, tandem breastfeeding remains a relatively under-researched and insufficiently discussed practice in routine maternal and child health counselling. Existing literature suggests that mothers may associate tandem breastfeeding with emotional continuity, sibling adjustment and prolonged breastfeeding. However, the practice may also raise practical and clinical questions, including maternal fatigue, sleep disruption, nutritional demands, feeding patterns of the older child, the feasibility of breastfeeding two children, family support, breastfeeding during pregnancy and concerns regarding maternal, fetal and infant well-being. These issues are particularly important because mothers’ decisions are not shaped only by biomedical information, but also by cultural expectations, family attitudes, professional advice and the availability of reliable counselling.

In contexts such as Türkiye, where breastfeeding is culturally valued but maternal behaviour is also strongly influenced by family norms and social expectations, tandem breastfeeding may be perceived in conflicting ways. Some mothers may view it as an extension of responsive motherhood, whereas others may consider it physically demanding, socially inappropriate or clinically uncertain. Therefore, examining mothers’ perceptions and decision-making processes may help healthcare professionals understand not only the emotional meanings attached to tandem breastfeeding, but also the practical and social conditions that facilitate or limit this practice.

### Aim of the work

This study aimed to explore mothers’ perceptions and decision-making processes regarding tandem breastfeeding in Türkiye. Specifically, the study sought to examine how mothers interpret tandem breastfeeding in relation to emotional bonding, maternal identity, practical feasibility, social norms, and professional support. The study did not aim to document the lived experiences of a defined sample of mothers practising tandem breastfeeding or to measure maternal, fetal, infant, or older-child health outcomes. Rather, it focused on mothers’ perceptions, beliefs, observed examples, anticipated concerns, and decision-making considerations.

## Methods

### Research design

This study is a qualitative research project based on a reflexive thematic analysis approach, conducted to examine how mothers perceive tandem breastfeeding and how these perceptions are shaped within emotional, social and health-related contexts. The study was reported in accordance with the COREQ (Consolidated Criteria for Reporting Qualitative Research) checklist, one of the international standards for reporting qualitative studies [8].

The research was conducted in Türkiye. Data were collected between January 2026 and March 2026. The study sample consisted of mothers with children aged 0–2 years who were familiar with or had heard about tandem breastfeeding. To obtain rich and diverse data, purposive sampling and snowball sampling methods were used in combination. A total of 21 mothers were included in the study. Recruitment was stopped at 21 mothers because informational redundancy was reached during the ongoing analysis. In the final interviews, mothers’ accounts repeated previously identified perceptions, concerns and decision-making considerations, and no substantially new codes or themes emerged. Therefore, the sample was considered sufficient to address the study aim.

The study focused on mothers’ perceptions of tandem breastfeeding rather than exclusively on mothers with current or past direct tandem breastfeeding experience. Therefore, mothers included mothers who were familiar with the concept of tandem breastfeeding and were able to discuss their views, beliefs, perceived benefits, concerns and decision-making considerations. This distinction was considered important during data analysis, as some accounts reflected lived or observed experiences, whereas others reflected anticipated concerns, social beliefs or hypothetical decision-making. Accordingly, the findings are presented as mothers’ perceptions and interpretations rather than as direct evidence of clinical outcomes or universal lived experience.

Because current or previous direct tandem breastfeeding practice was not systematically recorded as a categorical mother characteristic, the number of mothers with confirmed direct tandem breastfeeding experience could not be reported. Therefore, the findings are interpreted as perceptions, beliefs, observed examples and decision-making considerations rather than as confirmed lived experiences of tandem breastfeeding practitioners.

### Inclusion criteria

- Being a mother with at least one child aged between 0 and 2 years.
- Being familiar with or having heard about tandem breastfeeding.
- Being able to express perceptions, beliefs, observed examples, anticipated concerns or decision-making considerations related to tandem breastfeeding.
- Being able to communicate in Turkish.
- Voluntarily agreeing to participate in the study.

### Exclusion criteria

- Having communication difficulties that would prevent participation in an in-depth interview.
- Declining audio recording.
- Not providing informed consent.

### Data collection process

Data were collected through face-to-face, semi-structured in-depth interviews conducted with mothers in a private and comfortable setting. The interview guide and questions were developed by the researchers based on the current literature on tandem breastfeeding, maternal-child health, and breastfeeding practices (Table 1).

Each interview lasted approximately 30–45 min and was conducted by a female academic researcher who was not hospital staff and had no clinical, therapeutic, supervisory or institutional relationship with the mothers. The interviewer had no children and no personal experience of tandem breastfeeding; therefore, reflexive notes were maintained throughout data collection and analysis to minimize the potential influence of researcher assumptions. Written informed consent, including permission for audio recording, was obtained from all mothers before the interviews. Field notes were taken during and after each interview to document contextual and non-verbal information. Audio recordings were transcribed verbatim using Transkriptor software and transferred to Microsoft Word. Mothers were anonymized using unique codes (A1–A21), and all identifying information was removed.

Sampling and data collection continued until meaning saturation was judged to have been reached. After each interview, the researchers reviewed field notes and preliminary codes to identify whether new meanings, patterns or dimensions related to tandem breastfeeding were emerging. By the final interviews, no substantially new themes were identified, and the data were considered sufficiently rich to explain the main patterns in mothers’ perceptions. Saturation was discussed and agreed upon by the research team through repeated analytic meetings. In this study, saturation referred not simply to the repetition of codes, but to the point at which additional interviews no longer added meaningful analytical depth to the developing themes.

**Table 1:** Interview guide

• How do you define tandem breastfeeding, and what does this concept mean to you?
• What are your thoughts on continuing to breastfeed your older child when a new baby is born? If you have experienced this situation, was the older child breastfed during the pregnancy of the new baby? If so, did breastfeeding continue throughout the entire pregnancy or only during a specific period? How was the older child fed during this time: direct breastfeeding, expressed breast milk, or both?
• How do you think tandem breastfeeding affects the bond between the mother and her children?
• How does breastfeeding two children simultaneously influence your sense of motherhood and maternal competence?
• How do you think tandem breastfeeding may affect you physically?
• What kind of emotional experiences do you associate with tandem breastfeeding (e.g., happiness, stress, anxiety)?
• How do people around you (family, society) perceive tandem breastfeeding, and how does this influence you?
• What kinds of misconceptions or lack of information about tandem breastfeeding have you observed in society?
• How do you evaluate the support and information provided by healthcare professionals regarding tandem breastfeeding?
• Overall, what factors influence your decision about whether to practice tandem breastfeeding?

The semi-structured interview guide was developed by the research team after reviewing the literature on breastfeeding during pregnancy, tandem breastfeeding, maternal-child health and breastfeeding counselling. The draft guide was reviewed by two academics with expertise in maternal and child health and one researcher experienced in qualitative methodology. Based on their feedback, questions were revised to improve clarity, reduce leading wording and better capture mothers’ views on practical feasibility, social influences and professional support. A pilot interview was conducted with one mother who met the inclusion criteria. Following the pilot interview, minor wording revisions were made to improve comprehensibility; no major changes were required, and the pilot interview was not included in the final analysis.

### Data analysis

The data were analysed in accordance with the reflexive thematic analysis approach defined by Braun and Clarke [9]. During the analysis process, the interview transcripts were coded inductively using the MAXQDA 24 software. Codes were grouped into meaningful patterns in line with the researchers’ active interpretative role to form themes. Direct quotations from the mothers’ statements (A1–A21) were used to support each theme.

The interviews were conducted and audio-recorded in Turkish. Audio recordings were transcribed verbatim in Turkish using Transkriptor software, and the transcripts were checked and corrected by the researchers by comparing them with the original recordings. Data analysis was conducted using the Turkish transcripts. Selected quotations and relevant manuscript sections were then translated into English by the researchers and cross-checked against the original Turkish transcripts to ensure conceptual accuracy. Translation discrepancies were resolved through discussion and consensus.

Analysis moved beyond descriptive coding by examining how mothers’ perceptions were shaped by intersecting emotional, practical, cultural and professional influences. During theme development, the researchers compared accounts that framed tandem breastfeeding as emotionally meaningful with accounts that emphasised fatigue, uncertainty, social judgement or lack of professional guidance. This comparative process helped the team interpret tandem breastfeeding as a negotiated maternal decision rather than merely a feeding practice. Particular attention was paid to whether mothers were describing direct experience, observed examples, social beliefs or hypothetical concerns.

During coding and theme development, particular attention was paid to whether mothers’ statements appeared to reflect personal experience, observed examples, socially shared beliefs, or anticipated concerns. However, because direct tandem breastfeeding experience had not been systematically recorded at recruitment, these types of accounts could not be analysed as clearly defined mother subgroups. The analysis therefore focused on shared patterns of meaning across mothers’ perceptions and decision-making narratives rather than on comparisons between mothers with and without confirmed tandem breastfeeding experience.

### Trustworthiness

Trustworthiness was ensured using Lincoln and Guba’s criteria of credibility, transferability, dependability and confirmability [10]. Credibility was strengthened through in-depth interviews, verbatim transcription, researcher discussions and the use of direct quotations to support each theme. Transferability was supported by providing contextual information about mothers’ socio-demographic and obstetric characteristics and the Turkish socio-cultural context in which breastfeeding decisions were shaped. Dependability was enhanced by documenting the data collection, transcription, coding and theme development processes. Confirmability was supported through reflexive field notes, analytic memos and team discussions aimed at ensuring that interpretations remained grounded in mothers’ narratives rather than researchers’ assumptions.

### Researcher reflexivity

The research team consisted of academics and health professionals working in the fields of maternal and child health, midwifery and nursing. This disciplinary background may have sensitised the researchers to the importance of breastfeeding continuity and professional counselling. At the same time, it may have created assumptions that breastfeeding should generally be supported whenever possible. To address this, the researchers engaged in reflexive discussions throughout data collection and analysis. Field notes and analytic memos were used to record first impressions, potential assumptions and alternative interpretations.

The researchers did not have a prior therapeutic or care relationship with the mothers. During interviews, care was taken to avoid presenting tandem breastfeeding as either a recommended or discouraged practice. Instead, mothers were invited to describe their own perceptions, concerns and decision-making considerations. During coding and theme development, the team actively questioned whether interpretations were based on mothers’ accounts or on professional assumptions about breastfeeding. Divergent accounts, including cautious or negative views about tandem breastfeeding, were retained in the analysis to provide a balanced interpretation.

## Results

The findings should be interpreted as mothers’ perceptions, beliefs, observed examples, anticipated concerns, and decision-making considerations regarding tandem breastfeeding. Direct current or previous tandem breastfeeding experience was not systematically documented, and it was therefore not possible to distinguish mothers with confirmed lived experience from those discussing the practice hypothetically or on the basis of social knowledge and observation. Consequently, the themes presented below do not constitute an account of the lived experiences of tandem breastfeeding mothers and do not demonstrate the clinical effects, safety, feasibility, or outcomes of tandem breastfeeding.

An examination of the mothers’ socio-demographic and obstetric characteristics revealed that the ages of the 21 mothers included in the study ranged from 21 to 40, with the vast majority falling within the 31–40 age group. When the mothers’ employment status was assessed, it was determined that some were in employment whilst others were not. In terms of educational attainment, the majority of the mothers were university graduates; however, it was also found that some had completed secondary education or postgraduate studies. In terms of the number of births, it was found that the majority of mothers had experienced two or three pregnancies. When the planning status of the pregnancies was examined, it was observed that a significant proportion of the pregnancies were planned, whilst unplanned pregnancies were also present at a similar level. The age gap between children is mostly between 2 and 3 years, whilst in a smaller number of mothers, this gap was found to be 1 year or 4 years. When the mothers’ current breastfeeding status was assessed, it was found that approximately half were still breastfeeding, whilst the other half were not (Table 2).

**Table 2:** Mother characteristics

Characteristic	Mothers, *n* or summary
Total participants	21
Age range	21–40 years
Predominant age group	31–40 years
Education	Mostly university graduates; some secondary education and postgraduate education
Employment status	Both employed and unemployed mothers
Number of pregnancies	Mostly two or three pregnancies
Pregnancy planning status	Both planned and unplanned pregnancies
Age gap between children	Mostly 2–3 years; fewer mothers reported 1 or 4 years
Current breastfeeding status	Approximately half were currently breastfeeding
Tandem breastfeeding status	Not systematically recorded; participants’ accounts were analysed as perceptions, beliefs and decision-making considerations

Some mother characteristics are presented as summary information because these variables were not systematically recorded as categorical variables in the original mother information form. Therefore, these characteristics are reported descriptively and should be interpreted as contextual information rather than as variables for subgroup comparison

### Theme 1. The perceived meaning and significance of tandem breastfeeding

This theme reflects the meanings that mothers attributed to tandem breastfeeding. Mothers commonly associated the practice with maintaining the mother–child bond, avoiding abrupt weaning of the older child, meeting the perceived needs of two children, and preserving a sense of fairness between siblings. These accounts should be understood as mothers’ interpretations and perceived meanings rather than as confirmed outcomes reported by a clearly identified sample of mothers with direct tandem breastfeeding experience.

#### Sub-theme 1.1. The basic definition of tandem breastfeeding: breastfeeding two children at the same time

A significant proportion of mothers defined tandem breastfeeding directly and explicitly as ‘breastfeeding two children at the same time / during the same period’. However, some statements suggest that tandem breastfeeding is understood within a broader continuum—not merely as simultaneous breastfeeding, but as the continuation of breastfeeding during pregnancy and its continuation after birth. This finding suggests that while mothers have a basic understanding of the concept of tandem breastfeeding, they may not always possess deeper knowledge regarding its clinical, psychosocial, and care-related dimensions.

*“It brings to mind breastfeeding both my children at the same time.”* *(A1)*

*“It makes me think of breastfeeding the older child and the newborn at the same time.”* *(A2)*

*“It brings to mind breastfeeding during pregnancy and breastfeeding both children after birth.”* *(A9)*

#### Sub-theme 1.2. The desire to maintain the bond and not deprive the first child

Mothers perceived tandem breastfeeding as a potentially positive approach because they associated it with maintaining the bond established with the older child and avoiding an abrupt end to the breastfeeding relationship. In this context, tandem breastfeeding was interpreted by mothers as a form of care that may support perceived emotional continuity.

*“The mother’s feelings of guilt may diminish because she is not forced to wean her first child.”* *(A1)*

*“It can reduce sibling jealousy and eliminate the necessity of abruptly weaning the first child.”* *(A4)*

*“She may choose it to maintain* the bond with her first child and avoid depriving them.” *(A12)*

#### Sub-theme 1.3. Being able to cope with two children and finding peace of mind in motherhood

Mothers associated tandem breastfeeding with the perceived ability to meet the needs of two children and fulfil their maternal role. These accounts indicate that mothers linked tandem breastfeeding with a perceived sense of maternal competence.

*“A mother may feel at peace with her conscience by believing she is able to provide for both her children.”* *(A3)*

*“Believing that neither of her children is being neglected may bring the mother happiness.”* *(A18)*

*“A mother may feel capable by believing she is able to provide for both her children.”* *(A9)*

### Theme 2. The burden of tandem breastfeeding

This theme reflects the physical, emotional, and care-related burdens that mothers associated with tandem breastfeeding. Mothers perceived the practice as potentially involving fatigue, sleep deprivation, increased care demands, stress, and emotional ambivalence. Because direct experience was not systematically documented, these findings represent perceived or anticipated burdens and should not be interpreted as the prevalence or severity of difficulties experienced by mothers actively practising tandem breastfeeding.

#### Sub-theme 2.1. Physical exhaustion and physical burnout

Mothers perceived tandem breastfeeding as potentially physically exhausting and associated breastfeeding two children during the same period with increased energy demands and fatigue.

*“Having a second baby while the first child is still breastfeeding can be quite exhausting for the mother.”* *(A1)*

*“Constant contact*,* fatigue and sleep deprivation can be challenging for the mother.”* *(A4)*

*“I think the most significant challenge is physical fatigue.”* *(A6)*

#### Sub-theme 2.2. Emotional ambivalence: the coexistence of competence and happiness with anxiety and stress

Mothers associated tandem breastfeeding with both positive and negative emotional meanings. Some linked the practice with anticipated happiness and maternal competence, whereas others associated it with possible stress and anxiety.

*“It can be tiring for the mother at times; at other times*,* it can make her feel good. This depends somewhat on the mother’s personal experience.”* *(A1)*

*“A mother may feel a sense of pride at having been able to breastfeed both her children.”* *(A3)*

*“Due to the constant effort to maintain a balance*,* a mother may feel tired and stressed.”* *(A10)*

#### Sub-theme 2.3. The oscillation between empowerment and strain in the role of motherhood

Mothers perceived that tandem breastfeeding could have both potentially empowering and challenging meanings for the maternal role.

*“A mother may experience guilt and fatigue*,* but she may also feel strong and capable.”* *(A4)*

*“It can give a mother a sense of competence.”* *(A6)*

### Theme 3. Social perceptions

Mothers perceived that tandem breastfeeding was generally not viewed positively in their social environment and could be regarded as a non-conforming practice.

#### Sub-theme 3.1. Negative social perceptions and being viewed as non-conforming

Mothers perceived that tandem breastfeeding could be regarded as unnecessary or inappropriate within their social environments.

*“I think society views this as a practice that should not be done.”* *(A1)*

*“People might ask*,* ‘Is there really a need for this?”* *(A3)*

*“I think it is generally not viewed positively.”* *(A10)*

#### Sub-theme 3.2. Misconceptions and lack of information

Mothers perceived that information about tandem breastfeeding was limited in their social environment and that various misconceptions were commonly expressed.

*“I think there is a lack of information on this subject.”* *(A5)*

*“I see that some people in my circle hold misconceptions such as ‘breast milk is harmful to the younger child’.”* *(A7)*

*“People might think that the taste of the milk will change and that the older child will stop breastfeeding.”* *(A9)*

#### Sub-theme 3.3. Maintaining personal decisions on motherhood despite societal attitudes

Some mothers perceived tandem breastfeeding as a personal maternal decision that should be shaped primarily by the mother’s own values, circumstances and judgements, despite social attitudes.

*“In this matter*,* mothers should prioritise their own feelings rather than the opinions of others.”* *(A6)*

*“Whatever my surroundings think*,* she should prioritise what she believes to be right.”* *(A18)*

*“I do not think it is right for society to be so distant towards tandem breastfeeding.”* *(A10)*

### Theme 4. Limited professional support

This theme reflects mothers’ perceptions that professional information and counselling regarding tandem breastfeeding were limited, inconsistent, or difficult to access. These accounts represent mothers’ views of professional support and should not be interpreted as an objective assessment of healthcare professionals’ knowledge or clinical practice.

#### Sub-theme 4.1. Perceived limitations and cautiousness in professional counselling

Mothers perceived that healthcare professionals did not always provide clear, standardised or supportive information about tandem breastfeeding. However, mothers’ accounts also suggested that professional hesitancy should not be interpreted only as misinformation. In some cases, healthcare professionals may adopt a cautious approach because of uncertainty regarding maternal fatigue, nutritional demands, pregnancy-related concerns or limited clinical evidence. This finding indicates that mothers need balanced counselling that acknowledges both the possible benefits of breastfeeding continuity and the practical or clinical concerns that may arise in individual cases.

*“I think healthcare professionals generally take a negative stance on this issue.”* *(A1)*

*“I think healthcare professionals are not sufficiently aware of this issue either.”* *(A3)*

*“In most cases*,* breastfeeding of the older child is discontinued by mothers when a new baby is born.”* *(A6)*

#### Sub-theme 4.2. Mothers’ inability to access adequate information and support

Mothers perceived that access to adequate counselling and guidance regarding tandem breastfeeding was limited. This sub-theme reflects mothers’ views that information gaps may be related not only to social misconceptions but also to limited access to institutional counselling and professional support.

*“I think mothers are not getting enough information and support on this issue.”* *(A1)*

*“They can find support if they ask for it; however*,* this support may not always be easily accessible.”* *(A3)*

*“No*,* mothers are not receiving sufficient support.”* *(A10)*

#### Sub-theme 4.3. Perceived lack of support and uncertainty in maternal decision-making

Mothers perceived that limited information and professional support could contribute to uncertainty in maternal decision-making. Some mothers used cautious and conditional expressions such as ‘if the conditions were right’, ‘I would consider trying it first’ or ‘I would assess the process as it naturally unfolds’. These accounts suggest that mothers’ evaluations of tandem breastfeeding may be shaped not only by personal beliefs but also by perceived access to information, social support and professional counselling.

*“I would try to assess the process as it naturally unfolds.”* *(A1)*

*“If it were me*,* I would consider trying it first.”* *(A7)*

*“If the conditions were right*,* I would definitely want to try tandem breastfeeding.”* *(A18)*

## Discussion

This study explored mothers’ perceptions and decision-making regarding tandem breastfeeding in Türkiye. The findings indicate that mothers understood tandem breastfeeding as a maternal and child health decision shaped by emotional bonding, perceived fairness between siblings, anticipated physical and emotional burden, social norms, family attitudes, and access to professional guidance. The contribution of this study lies in showing how mothers make sense of tandem breastfeeding and the factors they consider when evaluating the practice.

However, the findings must be interpreted in light of a central methodological limitation. Direct tandem breastfeeding experience was not systematically recorded, and mothers with confirmed lived experience could not be distinguished from those expressing beliefs, expectations, observed examples, or hypothetical concerns. Therefore, this study should not be presented as an account of the lived experiences of tandem breastfeeding mothers. Rather, it offers insight into mothers’ perceptions and decision-making processes regarding tandem breastfeeding.

### The significance of tandem breastfeeding: bonding and maternal competence

This study found that mothers associated tandem breastfeeding with meeting the perceived needs of two children, maintaining emotional continuity and sustaining the mother–child bond. These findings indicate that mothers may interpret tandem breastfeeding not merely as a feeding practice, but also as a relational and maternal decision involving meanings of bonding, care and motherhood. However, because direct tandem breastfeeding experience was not systematically documented, these accounts should be understood as perceived meanings and expectations rather than as confirmed relational outcomes of tandem breastfeeding. The literature also emphasises that mothers’ perceptions of breastfeeding competence whilst tandem breastfeeding constitute an important area of research [5]. Furthermore, previous case-based evidence suggests that mothers who become pregnant while breastfeeding may experience uncertainty about whether to continue breastfeeding or wean, highlighting the need for individualized counselling when there is no contraindication to breastfeeding during pregnancy [11]. This finding should be interpreted with caution. Although mothers’ narratives suggest that emotional processes may be associated with breastfeeding behaviour, the present study cannot determine a causal relationship. In tandem breastfeeding, the older child is often beyond infancy and may obtain a substantial proportion of nutrition from complementary foods rather than breast milk. Therefore, breastfeeding behaviour in older children may also reflect comfort-seeking, attachment, habit, sibling dynamics, or a desire for maternal attention, rather than nutritional need alone. These alternative explanations indicate that tandem breastfeeding is shaped by multiple emotional, relational, developmental, and practical factors.

In this study, mothers associated the perceived ability to care for two children with feelings of relief and maternal competence. However, these accounts represent mothers’ interpretations and anticipated meanings and do not demonstrate that tandem breastfeeding objectively strengthens maternal competence. However, the literature indicates that the duration of breastfeeding decreases as pregnancy progresses [12].

### The burden of tandem breastfeeding: physical and emotional aspects

In the present study, mothers associated tandem breastfeeding with anticipated physical tiredness, increased care demands and emotional strain. Pregnancy-related and fetal outcomes were not a primary focus of the study and were not systematically assessed. Moreover, because direct breastfeeding during pregnancy or tandem breastfeeding experience was not recorded as a mother characteristic, the absence of pregnancy-related concerns in some accounts should not be interpreted as evidence regarding maternal or fetal safety. The findings therefore reflect perceived or anticipated burdens rather than clinically documented effects of tandem breastfeeding.

The study findings indicate that mothers perceive tandem breastfeeding as a physically demanding process. Breastfeeding two children simultaneously is thought to increase the mother’s energy requirements and intensify the care burden. Similarly, the literature reports that tandem breastfeeding is associated with physical fatigue, sleep deprivation and difficulties with time management [13]. However, mothers’ perceptions suggest that tandem breastfeeding may be understood not only in relation to anticipated physical strain but also as a practice associated with both positive and negative emotional meanings. Whilst some mothers associate this process with feelings of happiness, pride and competence, others report experiencing anxiety and stress [14]. Similarly, a study by Öztaş and Durmuş (2025) indicates that tandem breastfeeding involves an intense process of emotional interpretation for mothers. When these findings are considered together, it becomes clear that tandem breastfeeding is a complex experience for mothers, possessing both empowering and challenging aspects [15].

The present findings also suggest that feasibility is central to mothers’ decision-making. Tandem breastfeeding may be perceived positively in terms of its emotional meaning while also being viewed as potentially difficult to implement in daily life. Maternal fatigue, sleep deprivation, the older child’s feeding demands, the newborn’s feeding frequency, household responsibilities and the availability of family support may all influence whether a mother feels able to initiate or continue tandem breastfeeding. Therefore, counselling should not frame tandem breastfeeding only as a matter of maternal willingness or knowledge; it should also assess the mother’s physical capacity, support system, workload and individual family context.

### Social perceptions: cultural norms and misconceptions

In the study, mothers stated that tandem breastfeeding is generally regarded as a non-normative practice in society. The literature also indicates that some women hold the view that breast milk is intended solely for the younger infant [16]. This situation demonstrates that tandem breastfeeding behaviours are shaped not only by individual preferences but also by cultural norms and the social environment. Sökmen’s (2026) study reveals that breastfeeding during pregnancy and tandem breastfeeding behaviours are not shaped solely by individual knowledge and preferences; they are also shaped under the influence of cultural taboos, religious beliefs and the social environment. In particular, statements such as “breastfeeding during pregnancy is harmful”, “it negatively affects foetal development”, “breast milk could poison the baby”, or “breast milk is the right of the unborn baby”, which women acquire from their surroundings, generate a strong social perception despite lacking a scientific basis. Such beliefs reflect not only a lack of information but also the decisive role played by cultural discourses and norms, passed down from generation to generation, in shaping maternal behaviour [17].

Widespread social misconceptions may influence how mothers evaluate tandem breastfeeding and make decisions regarding whether to initiate or continue the practice. The literature indicates that, despite environmental pressures and negative societal attitudes, mothers continue to uphold their own parenting decisions during the tandem breastfeeding process [15]. The present findings are consistent with literature showing that breastfeeding decisions may be negotiated within social and cultural contexts. Mothers perceived the attitudes of people around them as potentially restrictive or negative, yet some emphasised that the final decision should reflect the mother’s own values, family circumstances, perceived child needs and approach to care.

### Limited professional support: evidence gaps, safety concerns, and counselling needs

In this study, mothers perceived that healthcare professionals did not always provide sufficient, clear or consistent information regarding tandem breastfeeding. These findings represent mothers’ perceptions of professional counselling and should not be interpreted as an objective assessment of healthcare professionals’ knowledge. Nevertheless, these perceptions are consistent with literature reporting information gaps and uncertainty among both mothers and healthcare professionals regarding breastfeeding during pregnancy and tandem breastfeeding [18]. In the study by Gül and Kılıçlı (2025), important implications for healthcare professionals are presented, and it is emphasised that mothers’ decisions regarding the tandem breastfeeding process are shaped not only by individual factors but also by family and societal influences. In this context, it is stated that breastfeeding counselling services should be developed to include tandem breastfeeding, and that healthcare professionals should be empowered to provide accurate, up-to-date and unbiased information on this subject. It is thought that, with increased awareness, tandem breastfeeding could become a more widely accepted practice [19].

The literature indicates that both mothers and healthcare professionals experience a significant lack of knowledge regarding breastfeeding during pregnancy and tandem breastfeeding, leading to confusion. In particular, uncertainties regarding the effects of breastfeeding during pregnancy on the mother, foetus and newborn increase anxiety levels in mothers and complicate the decision-making process [20]. In line with these findings, mothers in the present study perceived limited information and professional support as factors that could contribute to uncertainty in their decision-making regarding tandem breastfeeding. Some mothers used more cautious and conditional expressions such as “if the conditions are right”, “I would consider trying it first” or “I would assess the process as it naturally unfolds”.

A balanced interpretation is also needed when considering healthcare professionals’ cautiousness. While lack of knowledge and inconsistent counselling may contribute to mothers’ uncertainty, professional caution may also reflect legitimate concerns about maternal exhaustion, nutritional adequacy, pregnancy-related risks, fetal well-being, infant growth or the limited availability of high-quality evidence in some areas of tandem breastfeeding. Therefore, the findings should not be interpreted as suggesting that all caution is a misconception. Rather, they indicate the need for evidence-based counselling that distinguishes between unsupported myths and clinically relevant concerns requiring individual assessment.

### Implications for maternity, child health and breastfeeding counselling

Overall, the main message of this study is that mothers considering tandem breastfeeding may need balanced, individualised and evidence-based support. Mothers associated tandem breastfeeding with preserving the mother–child bond and supporting a perceived sense of fairness between siblings, while also associating it with anticipated fatigue, emotional strain, social judgement and uncertainty regarding professional guidance. These findings extend current understanding by showing that mothers may evaluate tandem breastfeeding not only as a feeding practice but also as a relational, practical and counselling-related decision. Because direct tandem breastfeeding experience was not systematically documented, these findings should inform understanding of maternal perceptions and counselling needs rather than clinical recommendations regarding the benefits, safety or feasibility of tandem breastfeeding.

The findings have implications for maternity care, child health and breastfeeding counselling by identifying the factors mothers may consider when evaluating tandem breastfeeding. Counselling may need to address not only breastfeeding technique but also maternal expectations, perceived fatigue, feeding routines, adjustment of the older child, family communication and access to reliable information. Healthcare professionals should provide balanced information that acknowledges mothers’ emotional motivations while also discussing practical feasibility and clinically relevant concerns. Such counselling should be individualised according to maternal health, pregnancy status, infant growth, nutritional needs, available family support and maternal preferences. However, the present findings should not be used to determine the clinical safety or effectiveness of tandem breastfeeding because these outcomes were not assessed.

These findings are important because tandem breastfeeding is closely related to pregnancy, postpartum care, breastfeeding counselling, maternal well-being and family-centred child health practice. By showing how social expectations, maternal ideals and limited professional guidance shape mothers’ perceptions of a breastfeeding practice that remains insufficiently addressed in routine care, this study provides qualitative evidence from the Turkish context.

### Limitations

The central methodological limitation of this study is that mothers’ current or previous direct tandem breastfeeding experience was not systematically recorded. Consequently, mothers with confirmed lived experience could not be distinguished from those expressing perceptions, expectations, socially shared beliefs, observed examples or hypothetical concerns. This substantially limits the extent to which the findings can be interpreted as representing the lived experience of tandem breastfeeding. Because direct experience could not be used as a mother characteristic, comparisons between mothers with and without confirmed tandem breastfeeding experience could not be conducted. Differences in perceived benefits, burdens, social responses, practical feasibility or professional support may therefore have remained unidentified. The findings were based on a qualitative sample of 21 mothers in Türkiye and are not intended to be statistically generalisable. Purposive and snowball sampling may also have resulted in the inclusion of mothers who were more willing to discuss breastfeeding or who held particularly strong views about tandem breastfeeding. These questions may have encouraged mothers to discuss anticipated benefits or concerns even when they did not have direct experience. Finally, the study was conducted within the socio-cultural context of Türkiye, where breastfeeding, motherhood, family involvement and intergenerational advice carry particular meanings. The transferability of the findings to other cultural and healthcare contexts should therefore be considered cautiously. Future studies should recruit clearly characterised groups of mothers with confirmed current or previous tandem breastfeeding experience and should systematically report the timing, duration and form of the practice.

## Conclusions

This study provides insight into mothers’ perceptions and decision-making processes regarding tandem breastfeeding in Türkiye. Mothers associated tandem breastfeeding with the perceived maintenance of the mother–child bond, avoidance of abrupt weaning and a sense of fairness between siblings. They also associated the practice with anticipated fatigue, increased care demands, emotional strain, social judgement and uncertainty regarding professional guidance. However, because direct tandem breastfeeding experience was not systematically recorded, the findings should not be interpreted as representing the confirmed lived experiences of mothers practising tandem breastfeeding or as evidence regarding its safety, feasibility, effectiveness or clinical outcomes. The primary contribution of this study is to identify the emotional, practical, social and professional considerations that may shape mothers’ perceptions and decisions regarding tandem breastfeeding. Healthcare professionals should provide balanced, culturally sensitive and individualised counselling that supports informed maternal decision-making without presenting tandem breastfeeding as either universally beneficial or universally problematic. Future studies should recruit clearly identified mothers with direct tandem breastfeeding experience and should prospectively examine maternal, infant, older-child and family outcomes.

## Data Availability

The datasets used and/or analyzed during the current study are available from the corresponding author on reasonable request.

## References

[1] Gila-Díaz A, Herranz Carrillo G, Cañas S, Saenz de Pipaón M, Martínez-Orgado JA, Rodríguez-Rodríguez P, de López ÁL, Martin-Cabrejas MA, Ramiro-Cortijo D, Arribas SM. Influence of maternal age and gestational age on breast milk antioxidants during the first month of lactation. Nutrients. 2020;12(9):2569. 10.3390/nu12092569. PMC7551022 32854220

[2] WHO. Infant and young child feeding. Geneva: WHO. 2023 Cited 2026 Mar 26. Available from: https://www.who.int/news-room/fact-sheets/detail/infant-and-young-child-feeding.

[3] Sinkiewicz-Darol E, Bernatowicz-Łojko U, Łubiech K, Adamczyk I, Twarużek M, Baranowska B, Skowron K, Spatz DL. Tandem breastfeeding: a descriptive analysis of the nutritional value of milk when feeding a younger and older child. Nutrients. 2021;13(1):277. 10.3390/nu13010277. PMC7835967 33478010

[4] American Academy of Family Physicians. Breastfeeding, family physicians supporting (position paper). Cited 2026 Mar 26. Available from: https://www.aafp.org/about/policies/all/breastfeeding-position-paper.html.

[5] Erdogan C, Turan T. Determination of breast milk insufficiency perceptions of tandem breastfeeding mothers: a case-control study. J Neonatal Nurs. 2023;29(1):50–3. 10.1016/j.jnn.2022.02.002.

[6] Coskun Ercelik H. [Nursing Approach for Breastfeeding in Tandem and Pregnancy]. Hacettepe Univ J Nurs. 2022;9(1):95–9. 10.31125/hunhemsire.1102089 . (in Turkish).

[7] Zengin H, Tiryaki Ö, Bafalı İO, Çınar N. [Opinions of Health Professionals About Breastfeeding during Pregnancy and Tandem Breastfeeding]. Ege Univ J Nurs. 2025;41(1):78–87. 10.53490/egehemsire.1405313 . (in Turkish).

[8] Tong A, Sainsbury P, Craig J. Consolidated criteria for reporting qualitative research (COREQ): a 32-item checklist for interviews and focus groups. Int J Qual Health Care. 2007;19(6):349–57. 10.1093/intqhc/mzm042. 17872937

[9] Braun V, Clarke V. Using thematic analysis in psychology. Qual Res Psychol. 2006;3(2):77–101. 10.1191/1478088706qp063oa.

[10] Lincoln YS, Guba EG. Naturalistic inquiry. Thousand Oaks (CA): Sage; 1985.

[11] Durmuş A, Coşkuner Potur D. [Breastfeeding at Two Different Ages: A Case Report]. Ordu Univ J Nurs Stud. 2022;5(2):223–8. 10.38108/ouhcd.939680 . (in Turkish).

[12] Sadıç E, Yılar Erkek Z. [Thoughts of Multiparal Pregnancy on Breastfeeding and Tandem Breastfeeding and Affecting Factors: Descriptive and Cross-Sectional Research]. Türkiye Klinikleri J Health Sci. 2025;10(3):658–69. 10.5336/healthsci.2025-109145 . (in Turkish).

[13] O’Rourke MP, Spatz DL. Women’s experiences with tandem breastfeeding. MCN Am J Matern Child Nurs. 2019;44(4):220–7. 10.1097/NMC.0000000000000534. 30888988

[14] Günaydın S, Dinç Kaya H, Yılmaz T, Duman R, Gür S. Mothers with tandem breastfeeding experience: A qualitative study. Early Child Dev Care. 2024;194(1):26–38. 10.1080/03004430.2023.2276659.

[15] Öztaş HG, Durmuş A. Women’s experiences and challenges with tandem breastfeeding: a qualitative study. Women Health. 2025;65(3):271–81. 10.1080/03630242.2025.2467742. 39965781

[16] In T, Koruk F. Determining breastfeeding behaviors during pregnancy and opinions on tandem breastfeeding among postpartum women in a high fertility region of Turkey. Niger J Clin Pract. 2024;27(3):401–7. 10.4103/njcp.njcp_739_23. 38528363

[17] Sökmen Y. Women’s breastfeeding experiences during pregnancy and thoughts on tandem breastfeeding: A cross-sectional study. Women’s Reproductive Health. 2026;1–11. 10.1080/23293691.2026.2634333.

[18] Serhatlıoğlu Göncü S, Yılmaz E. [What is tandem breastfeeding?]. IGUSABDER. 2020;12433–42. 10.38079/igusabder.658725. (in Turkish).

[19] Gül S, Kılıçlı A. It has not been easy, but it has been worth it: Experiences of tandem breastfeeding mothers: a qualitative research. J Hacettepe Univ Fac Nurs. 2025;12(1):26–37. 10.31125/hunhemsire.1502654.

[20] Doğancı P, Özsoy S. [Breastfeeding during pregnancy and tandem nursing]. Turkiye Klinikleri J Nurs Sci. 2019;11(2):190–9. 10.5336/nurses.2018-62206 . (in Turkish).

